# A Novel Sound-Absorbing Metamaterial Based on Archimedean Spirals

**DOI:** 10.3390/ma18225141

**Published:** 2025-11-12

**Authors:** Shasha Yang, Qihao Yang, Zeyu Du, Han Meng, Bo Song, Yuanyuan Li, Cheng Shen

**Affiliations:** 1 School of Mechanical Engineering, Nanjing University of Industry Technology, Nanjing 210023, China; 2016100849@niit.edu.cn (S.Y.); 15996263098@163.com (Q.Y.); 2 State Key Laboratory of Mechanics and Control of Mechanical Structures, Nanjing University of Aeronautics and Astronautics, Nanjing 210016, China; dzy0817@nuaa.edu.cn; 3 Jiangsu Province Precision Manufacturing Engineering and Technology Research Center, Nanjing 210023, China; 4 State Key Laboratory of Materials Processing and Die & Mould Technology, School of Material Science and Technology, Huazhong University of Science and Technology, Wuhan 430074, China; bosong@hust.edu.cn; 5 China Academy of Aerospace System and Innovation, Beijing 100176, China

**Keywords:** metamaterial, Archimedean spiral, sound absorption, transfer matrix method, experiment validation

## Abstract

Inspired by the concept of antennas in electromagnetics, this study proposes a novel acoustic metamaterial using Archimedean spiral structures. Unlike traditional resonant absorption structures, the present structure does not rely on resonant cavities but consists of multiple channels bent according to specific geometric parameters. The absorption mechanism is attributed to the combination of Fabry–Pérot (FP) resonance and viscous loss effects at waveguide boundaries. A theoretical model based on the transfer matrix method has been established and validated through numerical methods. Furthermore, the present study investigated the relationship between absorption performance and geometric parameters through theoretical analysis and numerical simulations, achieving efficient absorption across a wide frequency range and at low frequencies by adjusting these parameters. Additionally, samples have been fabricated using additive manufacturing techniques and experimental validation confirmed the accuracy of the theoretical and numerical simulations. The structure designed in this paper is expected to be applied to the engineering field with the need of broadband sound absorption.

## 1. Introduction

As urbanization accelerates, urban industrial noise, traffic noise, and noise from everyday elements such as elevators, transformers, and central air conditioning systems are significantly increasing [1,2]. There is an urgent need to develop and apply compact low-frequency, broadband materials or structures to meet the growing demand for noise reduction. Traditional noise reduction methods, such as porous materials [3], perforated structures [4], acoustic blankets with mass-loaded layers [5], and gradient-refractive index materials [6], have been widely used to mitigate noise pollution. Although these methods are effective in managing noise in the mid to high frequency range, their application is limited due to the requirement for substantial thickness.

Acoustic metamaterials offer the possibility of manipulating sound waves beyond the diffraction limit. Early research focused on negative parameter materials based on local resonance [7], while transformation optics theory has given rise to concepts such as acoustic cloaks. Cummer and Alù [8] outline the principles and potential of acoustic metamaterials in controlling sound waves, including concepts like negative refraction and cloaking. The theoretical framework of acoustic metamaterials (and their two-dimensional counterparts and acoustic meta-surfaces) originated from electromagnetic analogs, enabling unprecedented control over sound wave propagation through tailored reflection and transmission modulation [9]. These engineered structures further demonstrate potential for acoustic cloaking [10] by redirecting incident waves around objects.

In noise control applications, metamaterials have garnered significant attention due to their capacity to achieve near-perfect low-frequency sound absorption [11,12,13,14,15] at subwavelength thicknesses—a feat unattainable with conventional materials [16,17,18]. For example, Ciaburro et al. [19] investigated sound attenuation of an acoustic barrier made with metamaterial, and it was found that the ability to deflect acoustic waves is due to the geometric effects and periodic arrangement of the structures. Recently, Sanchez-Dehesa et al. [20] combined the surface of 2D Helmholtz resonators and a 2D sonic crystal to realize a metasurface for sound absorption over a broad range of wave incidence angles. Among prevalent designs, spatially coiled metamaterials stand out for their compact geometries and exceptional low-frequency absorption performance, leveraging coiled air channels to extend acoustic paths within limited volumes. This absorption arises from synergistic mechanisms including the following: localized resonance in subwavelength unit cells; the emergence of negative effective mass density or bulk modulus; critical coupling for impedance matching; and Fabry–Pérot resonances within coiled cavities [21,22,23,24]. Despite their subwavelength advantages, the reliance on resonant phenomena introduces inherent trade-offs: strong frequency dispersion and narrow operational bandwidth, which remain fundamental challenges for practical implementations.

To address these limitations, a common approach employs arrays of resonators operating at varied frequencies, broadening the bandwidth via combined local and non-local resonant effects. The introduction of porous matrices into resonant architectures significantly enhances absorption bandwidth, whereas inverse designs improve low-frequency dissipation in porous systems by efficiently entrapping and attenuating sound energy [25,26]. Integrating complementary resonance mechanisms—such as microperforated panels, Helmholtz resonators, and Fabry–Pérot cavities within unified metastructures—enables subwavelength-scale broadband absorption. Theoretical frameworks modeling effective medium properties have further advanced broadband elastic wave absorption [27]. Recent progress extends the Rozanov thickness–bandwidth tradeoff principle (originally from electromagnetics) to acoustic metamaterials, permitting the optimization of multi-resonator configurations for targeted sound-absorbing performance.

**Figure 1:**
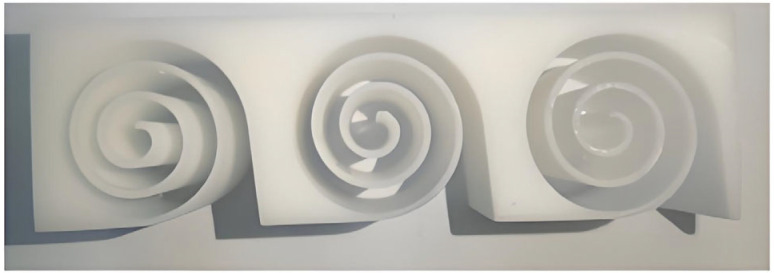
Photograph of Archimedean spiral structure sample.

So far, there are mainly two principles for realizing deep sub-wavelength sound absorption structures. One is the membrane structure proposed by Mei et al. [28] and the other is the space-coiling structure. Under the same sound propagation path length, space-coiling significantly reduces the thickness of the structure. Cai et al. [29] combined the principles of surface perforation and space-coiling to construct a new Helmholtz resonator, achieving effective low-frequency sound absorption under sub-wavelength thickness, but this method is more suitable for noise absorption at a single frequency. To improve this design, Kong et al. [30] proposed an ultra-thin sound-absorbing spiral metasurface with perforated partitions using recessed necks, achieving multi-modal sound wave absorption. Duan et al. [31] designed a multi-chamber parallel spiral structure, achieving excellent performance with an absorption coefficient greater than 0.8 within a 320 Hz bandwidth. While contemporary acoustic metamaterial research predominantly targets narrowband quasi-perfect absorption at specific low frequencies or employs multi-resonator arrays for bandwidth expansion, a critical challenge persists in designing a monolithic resonator capable of matching/exceeding the broadband performance of multi-unit systems within subwavelength dimensions. Drawing parallels to spiral antennas in electromagnetic theory, this work pioneers an acoustic metamaterial leveraging Archimedean spiral coiled cavities (ASCCs) (as shown in Figure 1) for broadband low-frequency dissipation. We establish a predictive absorption model via the transfer matrix method, validated against finite element simulations. Theoretical and numerical analyses elucidate the underlying dissipation mechanisms and parametric dependencies, demonstrating that strategic geometric tuning of the ASCC unit enables high-efficiency absorption across an extended low-frequency spectrum without requiring cascaded resonators.

## 2. Theoretical Model

**Figure 2:**
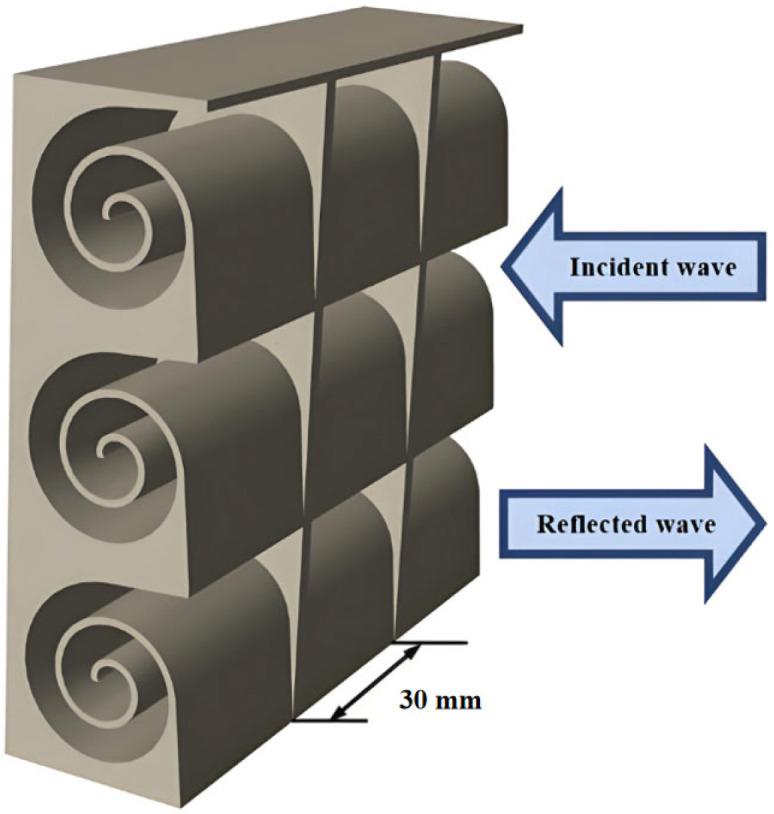
A schematic representation of incoming and reflected sound.

In general, the sound absorption coefficient of an acoustic absorption structure with a rigid backing can be determined by calculating its surface acoustic impedance, as shown in Figure 2. The core advantage of this technology lies in its ability to expand the sound absorption bandwidth through the spiral channel structure while maintaining structural compactness. It is suitable for scenarios with limited space and requiring strict acoustic isolation including architectural soundproof cabins, anechoic chambers, industrial equipment enclosures, and so on.

Below, the surface impedance of the structure is computed using the transfer matrix method (TMM). The TMM model is mainly based on the following assumptions: 1. the acoustic wave propagates in the form of a local plane wave within the infinitesimal segment; 2. the radial energy loss and the influence of higher-order modes can be ignored. In fact, the TMM is widely applicable to the frequency-domain analysis of chain structures (such as pipes and spiral channels) and can efficiently handle one-dimensional wave propagation problems. For instance, Liu et al. [32] designed a ventilation broadband sound absorber based on Archimedean spiral units. They used the transfer matrix method to analyze the sound pressure–velocity transfer relationship between adjacent spiral units and calculated the overall structure’s sound absorption coefficient.

Define the two Archimedean spiral curves that compose the helical waveguide as follows:

(1) $$\begin{array}{l} r_{1}=a_{1}+b\theta ,\;0\leq \theta \leq k_{1}\pi \\ r_{2}=a_{2}+b\theta ,\;0\leq \theta \leq k_{2}\pi \end{array}$$

**Figure 3:**
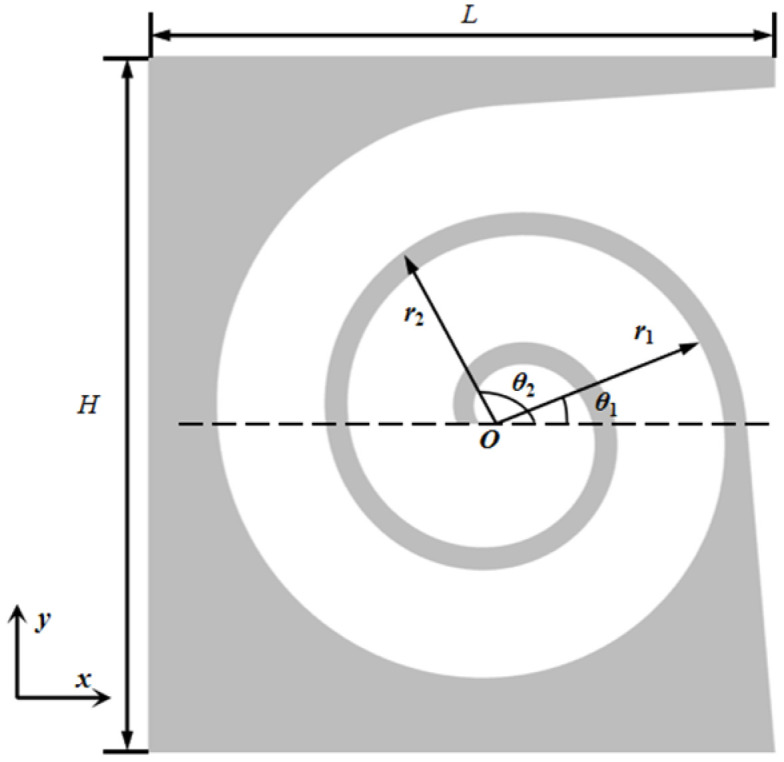
The cross-section of ASCC in the *xy* plane.

**Figure 4:**
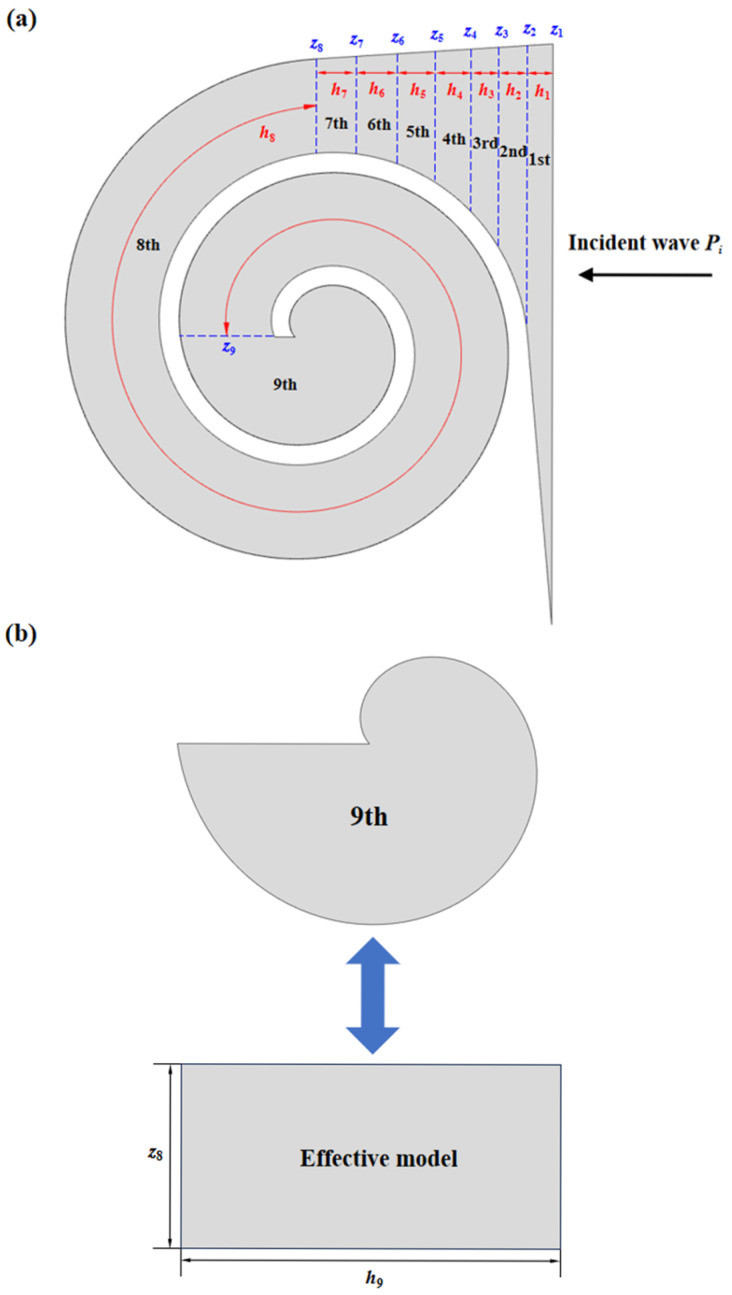
(**a**) Schematic of the transfer matrix method; (**b**) effective medium model of the third section.

The internal air domain of the structure can be divided into three main sections: The first section is the region with a gradual change in channel dimensions. To reduce computational effort while maintaining accuracy, this section is divided into seven smaller parts, labeled 1st to 7th in Figure 3 and Figure 4, with cross-sectional dimensions of *s*_i_ and cross-sectional impedance of *z*_i_. The second section is the helical part of the channel with a constant dimension of *s*_8_. The third section is the irregular portion at the end of the channel. The transfer matrix for the internal section of the *i*-th is given by:

(2) $$\left[\begin{array}{c} p_{i}\\ u_{i} \end{array}\right]=\left[\begin{array}{cc} \cos\left(k_{0}h_{i}\right) & j\frac{\rho_{0}c_{0}}{s_{i}}\sin\left(k_{0}h_{i}\right)\\ j\frac{s_{i}}{\rho_{0}c_{0}}\sin\left(k_{0}h_{i}\right) & \cos\left(k_{0}h_{i}\right) \end{array}\right]\left[\begin{array}{c} p_{i+1}\\ u_{i+1} \end{array}\right]$$

where *u* represents the volumetric velocity and *ρ*_0_ = 1.21 kg/m^3^ and *c*_0_ = 343 m/s denote the density and speed of sound in air, respectively. The normal acoustic impedance of the *i*-th section can be expressed as:

(3) $$z_{i}=\frac{-j\rho_{0}c_{0}z_{i+1}s_{i}\cot\left(k_{0}h_{i}\right)+{\left(\rho_{0}c_{0}\right)}^{2}}{z_{i+1}s_{i}^{2}-j\rho_{0}c_{0}s_{i}\cot\left(k_{0}h_{i}\right)}$$

For the helical section of the channel cross-section, it can be equivalently represented as a rectangular channel with dimensions *z*_8_ and *h*_8_, where *h*_8_ is the length of the rectangular channel, it can be expressed as the following:

(4) $$h_{8}=\frac{\int_{0}^{\left(k_{1}-2\right)\pi }\left[\frac{r_{1}\left(2\pi \right)+r_{2}\left(0\right)}{2}+b\theta \right]d\theta }{s_{8}}$$

For the irregular region at the end, it can be simplistically represented as a rectangle with the same area. The dimensions of the rectangle are *z*_8_ and *h*_9_. The area of the irregular region *A*_9_ and the equivalent channel length *h*_9_ can be expressed as:

(5) $$A_{9}=\int_{0}^{2\pi }\frac{r_{1}^{2}\left(\theta \right)}{2}d\theta ,h_{9}=\frac{A_{9}}{s_{8}}$$

The end of the waveguide is a rigid boundary, and its impedance is considered to be infinite. Therefore, *z*_9_ can be represented as follows:

(6) $$z_{9}=\frac{-j\rho_{0}c_{0}\cot\left(k_{0}h_{9}\right)}{s_{8}}$$

Therefore, the reflection coefficient *R* and the absorption coefficient α of the structure can be computed as follows:

(7) $$R=\frac{s_{1}z_{1}-\rho_{0}c_{0}}{s_{1}z_{1}+\rho_{0}c_{0}},\alpha =1-{\left|R\right|}^{2}$$

Notably, our primary objective was to achieve sound absorption in the broadband frequency domain. The spiral channel functions primarily as an acoustic resonator. The total spiral path length *L* is designed to be approximately a quarter-wavelength (λ/4) of the target resonant frequency. This *L* is determined by the number of turns, *N*, and the gap between channels g (i.e., *L* ≈ 2π*RN* for an idealized spiral, where *R* is the average radius). The width and depth of the channel were chosen to ensure a high aspect ratio, which suppresses higher-order modes and maintains the validity of the plane-wave assumption within our frequency range of interest. The outer diameter was constrained by practical application size limits.

## 3. Finite Element Model

The Finite Element Method (FEM) is an effective numerical technique for the acoustic prediction of complex geometric structures. In this study, COMSOL Multiphysics 6.1 finite element software is utilized for simulation.

Using the built-in meshing tool in COMSOL Multiphysics, we created a series of meshes with increasing refinement. These were based on the software’s predefined mesh levels, such as ‘Coarser’, ‘Normal’, ‘Fine’, ‘Finer’, and ‘Extra Fine’. We established a convergence criterion so that the simulation results can be considered converged when the change in the key indicator between two consecutive mesh levels is less than a predefined threshold (1% in present work). In addition, sensitivity analysis has been performed on the key parameters to evaluate the robustness of our design and to identify the dominant geometric parameters affecting acoustic performance. To accurately model wave propagation, the maximum element size must be significantly smaller than the shortest wavelength (λ_min_) corresponding to the highest frequency of interest. We followed the widely accepted rule of thumb, using at least five to six mesh elements per wavelength. The selection of boundary conditions in our model strictly emulates the physical environment of a standard normal-incidence sound absorption measurement (akin to a test in an impedance tube).

**Figure 5:**
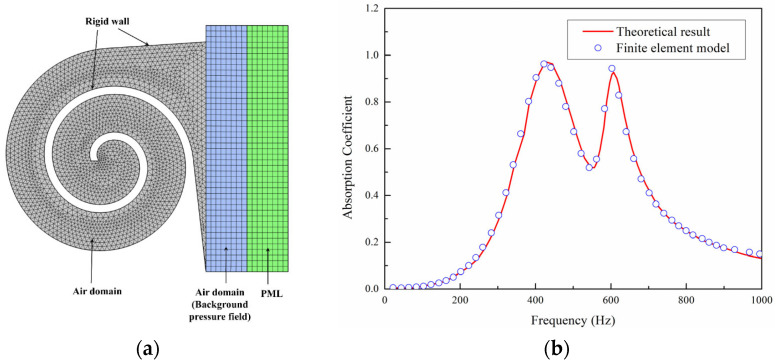
(**a**) The cross-section of the FE model. (**b**) Test case for finite element model.

Specifically, a model is established in the pressure acoustic domain, as shown in Figure 5a, to solve the linearized continuity equation, Navier–Stokes equations, and energy equations. Given that the stiffness of the structure is significantly greater than that of the air, only the medium within the structure is considered in the model, with a hard acoustic boundary condition applied at the external boundary, indicating zero acceleration and velocity at the interface. The air domain above the structure serves as the background acoustic field, providing an incident sound pressure of 1 Pa. A perfectly matched layer (PML) is applied above the background acoustic field to completely absorb scattered sound waves, ensuring that reflections at the boundary between the PML and the acoustic field do not affect the accuracy of the calculation. The structure domain uses a free triangular mesh, while the mesh for the air domain and PML is obtained by boundary layer sweeping. The computational frequency range is constrained by the plane wave assumption, and the cutoff frequency is given by Equation (8):

(8) $$f_{c}\leq \frac{1.84c_{0}}{2\pi R}$$

Here, *c*_0_ is the speed of sound in air and *R* is the geometric dimension of the structure’s cross-section, respectively. The impedance *Z*_s_ at the sound wave incidence interface and the absorption coefficient α can be obtained from Equation (9):

(9) $$Z_{s}=\frac{P_{in}}{u_{in}},\alpha =1-{\left|\frac{Z_{s}-\rho_{0}c_{0}}{Z_{s}+\rho_{0}c_{0}}\right|}^{2}$$

The complex sound speed *c* = *c*_0_ (1 + 0.05*j*) is used to account for the effects of damping, where 0.05 represents the damping loss factor, which approximately simulates the thermal viscous losses of the structure.

To further demonstrate the effectiveness of the present simulation method, we have added a test case. The research object is an origami-based acoustic metamaterial and the basic principle of sound absorption is mainly the Helmholtz resonator. Detailed theoretical results can be found in reference [12], which has also provided a theoretical model to validate numerical results. As shown in Figure 5b, the results show that the simulation results are in good agreement with the theoretical predictions, which to some extent indicates the effectiveness of the simulation.

## 4. Results and Discussion

### 4.1. Sound Absorption Performance of the ASCC

**Figure 6:**
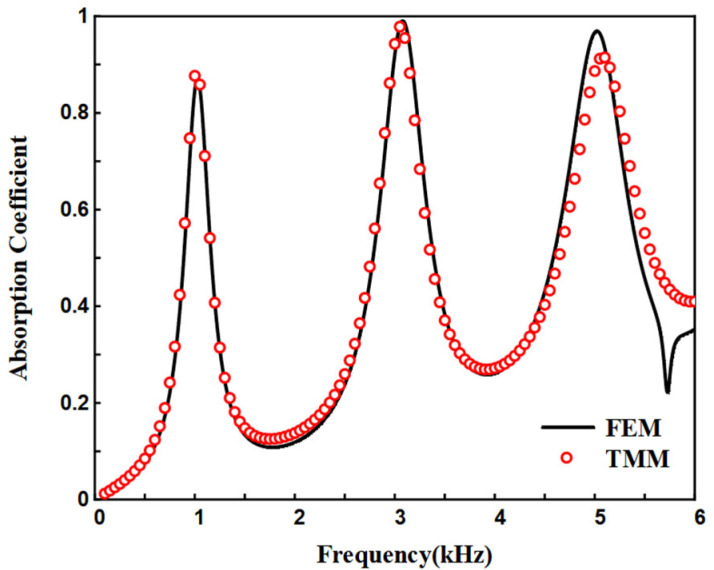
Sound absorption coefficients of the ASCC.

**Figure 7:**
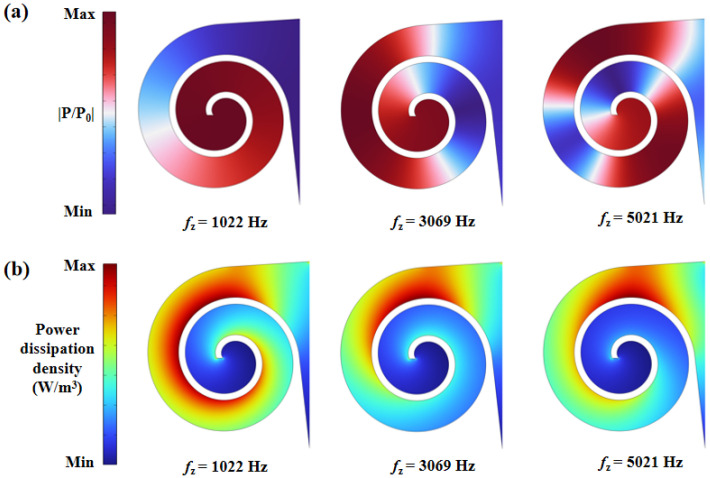
(**a**) Sound pressure; (**b**) total power density at absorption peaks.

Figure 6 shows the sound absorption coefficients of the structure calculated using both the FEM and TMM. It is evident that the results from both methods are in good agreement, validating the accuracy of the theoretical model. However, discrepancies between the two methods are observed in the high-frequency range of 4000 Hz to 6000 Hz. This is attributed to the complex propagation modes of high-frequency sound waves within the waveguide, which are not simply plane waves. The transfer matrix method assumes plane wave propagation in the waveguide, leading to discrepancies. The quarter-wavelength resonance generated by ASCCs results in high sound absorption at the peak values. Figure 7a presents the sound pressure maps at the three peak frequencies, corresponding to three different resonance modes. The ASCC waveguide length can be determined from the formula and for the resonance frequencies of 1022 Hz, 3069 Hz, and 5021 Hz, the waveguide length is an odd integer multiple of their quarter-wavelengths, satisfying the equation *L* = *n*·*λ*_i_/4. Figure 7b displays the total power density of the plane waves, reflecting the sound energy dissipation. It can be observed that at the peak absorption frequencies, sound energy dissipation is mainly concentrated on the inner wall near the waveguide’s pole. For 1022 Hz, the energy dissipation is relatively uniform, while at 3069 Hz and 5021 Hz, energy dissipation is primarily concentrated at the front section of the waveguide, focusing on the end where the waveguide’s size gradient transitions.

### 4.2. Influence of Radial Growth Rate B

**Figure 8:**
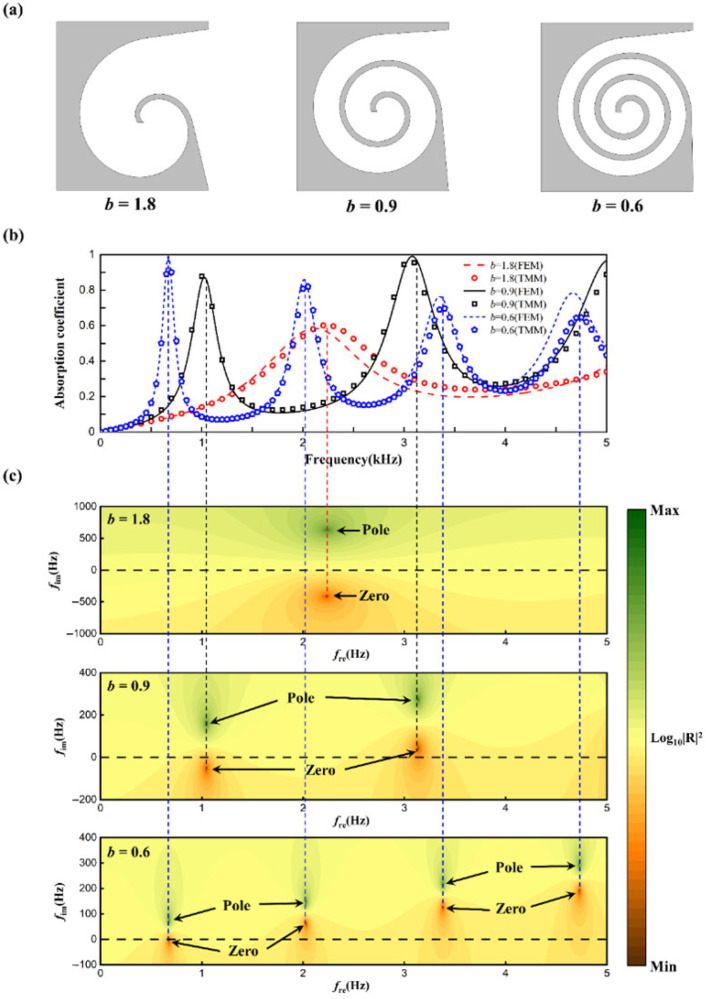
(**a**) The cross-section; (**b**) sound absorption coefficients. (**c**) Reflection coefficient in the complex frequency plane with different values of (**b**).

The radial growth rate characterizes how the spiral radius changes with angle. This section investigates the effects of three different radial growth rates on the sound absorption performance of the structure. To ensure a certain spatial efficiency, the spiral rotation angle is smaller when the radial growth rate is high compared to when it is low. It can be inferred that a slower radial growth rate results in a longer waveguide with a smaller cross-sectional area, while a faster growth rate leads to a shorter waveguide with a larger cross-sectional area. The theoretical model based on the transfer matrix method and the finite element model were used to calculate the sound absorption coefficients of the structure, with results shown in Figure 8. Both methods agree well across the frequency range, with minor discrepancies at high frequencies, consistent with previously explained patterns. For *b* = 1.8, the structure exhibits a lower absorption peak at 2234 Hz; for *b* = 0.9, there are three absorption peaks at 1022 Hz, 3069 Hz, and 5021 Hz, with the highest peak at 3069 Hz reaching 0.98; for *b* = 0.6, four absorption peaks are observed at 677 Hz, 2013 Hz, 3353 Hz, and 4667 Hz, with the highest peak at 677 Hz reaching 0.998. The number of absorption peaks varies due to different waveguide lengths; longer waveguides produce more absorption peaks.

To further study the mechanism by which the radial growth rate affects absorption, a graphical method was employed to examine the reflection coefficients in the complex frequency plane. By introducing an imaginary frequency *f_i_*, where *f* = *f_r_* + *if_i_* (with *f_r_* as the real frequency) and substituting the complex frequency into the reflection coefficient formula, the complex frequency reflection coefficient plots were obtained, as shown in Figure 8. Green and red points denote the extremal values of the complex reflection coefficients, called zeros and poles, respectively. In a lossless acoustic system, zeros and poles are symmetrically distributed around the real axis (black dashed line). When structural losses are considered, zeros and poles shift along the positive imaginary frequency axis. For *b* = 1.8, the zeros and poles shift slightly, resulting in a single low absorption peak due to the system being in an underdamped state. For *b* = 0.9, the structure is underdamped at 1022 Hz and overdamped at 3069 Hz. The zeros are closer to the real axis at these frequencies, leading to higher absorption peaks, with the peak at 3069 Hz being higher due to the zero being closer to the origin compared to 1022 Hz. For *b* = 0.6, the zero at 677 Hz falls on the real axis, achieving perfect absorption with a peak value of 0.998. As the frequency increases, the system becomes increasingly overdamped, resulting in lower absorption peaks at higher frequencies.

### 4.3. Influence of Initial Angle θ_0_

**Figure 9:**
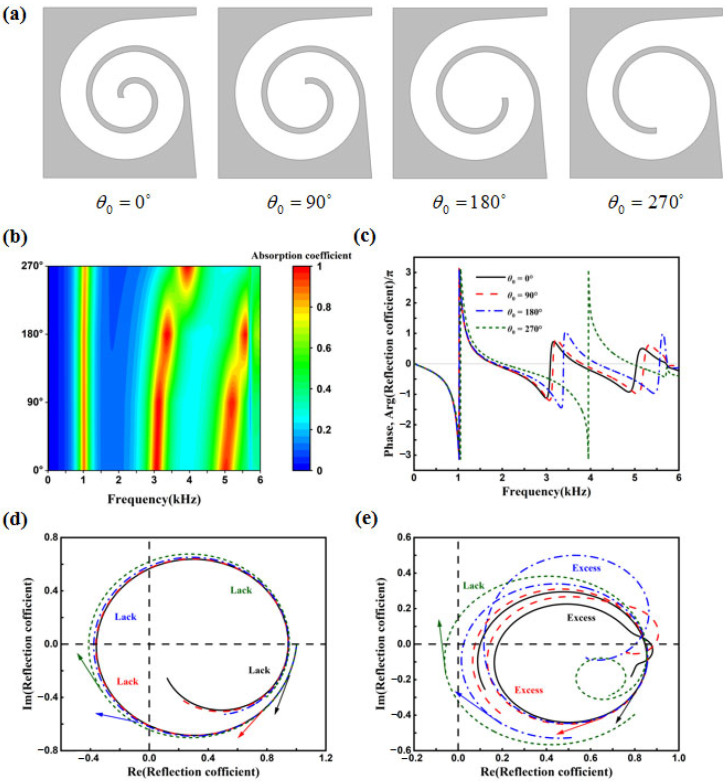
(**a**) The cross-section; (**b**) sound absorption coefficients. (**c**) Phase variation in the reflection coefficient; (**d**) Argand diagram for 0–3000 Hz; (**e**) Argand diagram for 3000–6000 Hz.

For a given Archimedean spiral curve, the starting angle affects the length of the spiral structure. As illustrated in Figure 9a, the 2D schematic diagrams for starting angles of 0°, 90°, 180°, and 270° are shown. The length of the bent waveguide decreases with an increase in the starting angle, while the volume of the end cavity gradually increases. Figure 9b presents the sound absorption coefficient maps for different starting angles in the frequency range of 0–6000 Hz. As the starting angle increases, the second peak of sound absorption shifts to higher frequencies. The second absorption peaks for starting angles of 0° and 90° are relatively close, whereas for 180° and 270°, there is a notable deviation. This deviation is attributed to the fact that although the gradient of the starting angle change is 90°, a larger angle causes more significant variations in waveguide length, thereby affecting the second absorption peak. A similar effect is observed for the third absorption peak. This section also examines the phase variation in the reflection coefficient on the complex plane and the Argand diagram, as shown in Figure 9c.

At resonant frequencies, the phase of the reflection coefficient exhibits abrupt changes, resulting in high sound absorption. The Argand diagram is commonly used to analyze the structural resonant state and changes in amplitude and phase upon reflection. The Argand diagram of the reflection coefficient appears as a curve rotating around the origin. At 0 Hz, the coordinate is at one on the real axis, and as the frequency increases, the curve rotates clockwise. Without considering losses, the Argand diagram is a circle with a radius of 1. When structural losses are considered, the trajectory becomes an elliptical path around the origin, enclosed within a circle of radius 1, shifting along the real axis. As losses increase, the diameter of the circumscribed circle of the ellipse decreases and the ellipse shifts towards the positive axis. The diameter of the circumscribed circle is inversely proportional to the loss. If the loss exceeds the critical coupling requirement, the trajectory does not include the origin. When the trajectory includes the origin, it indicates insufficient loss. Figure 9d shows the Argand diagram for 0–3000 Hz, revealing that at low frequencies, all four structures exhibit insufficient loss, with absorption peaks of 0.872, 0.869, 0.859, and 0.832, respectively. Figure 9e for 3000–6000 Hz indicates that structures with starting angles of 0°, 90°, and 180° have excessive loss, with absorption peaks of 0.988, 0.992, and 0.999, respectively, while the structure with a starting angle of 270° has insufficient loss, with an absorption peak of 0.997. Notably, for a starting angle of 180°, the curve is very close to the origin at a frequency of 3370 Hz. Although the system impedance does not perfectly match the air impedance, the absorption peak is 0.999 (approximately one), achieving near-perfect absorption.

## 5. Experimental Verification

**Figure 10:**
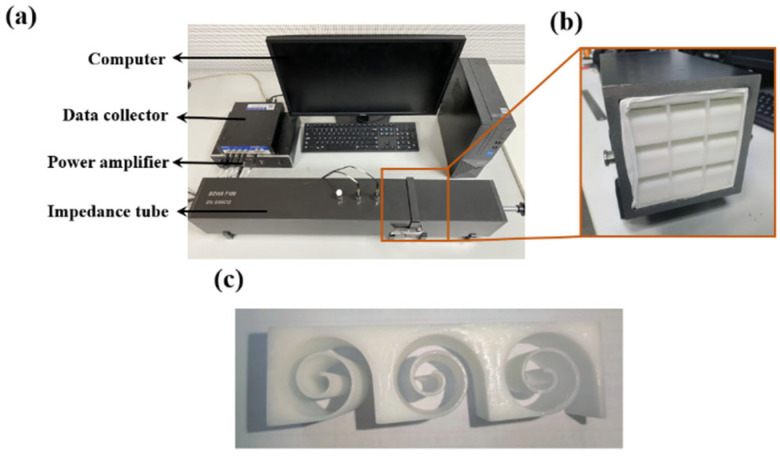
(**a**) Experimental facility; (**b**) sample positioned in impedance tube. (**c**) Prepared sample.

To further validate the accuracy of the theoretical and simulation calculations, experiments were conducted using the SW422 (BSWA Technology, Beijing, China) impedance tube, as shown in Figure 10a. Acoustic absorption tests were performed on Archimedean spiral metamaterials using the VA-Lab testing system and the transfer function method, as depicted in Figure 10. To minimize the variability in experimental results, an average of 12 experimental data sets were taken. The structures were 3D printed using white resin, with overall dimensions of 100 mm × 100 mm × 30 mm. For this rectangular waveguide, the cut-off frequency *f*_*c*,*m*,*n*_ of mode (*m*, *n*) is given by the following formula $f_{c,m,n}=\frac{c}{2}\sqrt{{\left(\frac{m}{a}\right)}^{2}+{\left(\frac{n}{b}\right)}^{2}}$. Substituting the data yields 1715 Hz. The frequency range we measure is 200 Hz-1600 Hz, which satisfies the plane wave assumption. Three samples are manufactured under the same controlled conditions and only one of them is shown in Figure 10c for brevity.

The following actions have been taken to ensure dimensional accuracy and material consistency of the 3D-printed samples. Firstly, the digital model (STL file) of the spiral structure has been carefully checked within the slicing software to ensure the toolpath generation accurately reflected the design’s dimensions. After printing, the samples are carefully cleaned to remove any support material. Key dimensions, including the outer diameter, the width of the spiral channel at multiple locations, and the wall thickness between channels, were physically measured using a digital caliper (with a resolution of 0.01 mm). All samples have been printed using the same batch of PLA filament from a single manufacturer to minimize variations in raw material properties. The printing process has been rigorously standardized: the same 3D printer model, nozzle diameter, layer height, printing temperature, and infill density (100% solid to ensure acoustic rigidity) are maintained for every sample. Generally, large-scale production of Archimedes spiral channel broadband acoustic absorbers is feasible, but it requires specific technical pathways and optimization strategies. Three-dimensional printing technologies (e.g., fused deposition modeling or stereolithography) have been successfully used to fabricate complex acoustic structures, offering advantages such as high design freedom, strong customizability, and the ability to produce geometries (e.g., spiral channels or microporous skeletons) difficult to achieve with traditional methods.

Notably, ensuring that the sample is sealed is a very crucial and common issue in impedance tube testing. During the sample installation process, a thin layer of raw material belt should be evenly and continuously applied around the edge of the sample. The raw material belt can perfectly fill microscopic gaps and is easy to apply and clean. Additionally, when inserting the sample into the sample holder and pushing it into the impedance tube, ensure uniform pressure from the back. Avoid tilting the sample in the tube, which could cause a gap on one side and compression on the other. After installing the sample, a transfer function measurement should be conducted first. Observe the amplitude and phase of the transfer function between the two microphones. A well-sealed sample has a standard standing wave field in its sound field. If an abnormally high absorption coefficient is found in the low-frequency range (such as close to 1) or if the transfer function curve shows abnormal fluctuations, there is a high possibility of leakage.

**Figure 11:**
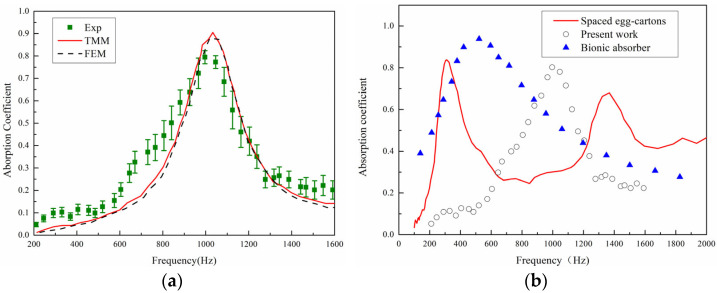
(**a**) Sound absorption coefficients of samples. (**b**) Comparison with others.

The accuracy of the theoretical model was validated by comparing the acoustic absorption coefficient curves obtained from the ASCC theoretical model, simulation results, and experimental results in the frequency range of 200–1600 Hz, as shown in Figure 11a. The experimental results shown are average results with error bars of three tested specimens. It is evident that the absorption curves obtained from the three methods exhibit the same trend, with a similar number of absorption peaks and peak frequencies that are very close, thus confirming the predictive accuracy of the theoretical model. Discrepancies between the results may stem from several factors: firstly, sample fabrication errors, such as structural dimension inaccuracies due to the precision limitations of 3D printing; secondly, experimental testing errors, such as gaps between the sample and the inner wall of the impedance tube and the difficulty in achieving ideal normal incidence of plane waves during the experimental process. Experimental measurements of density and damping have not been conducted in this work and internal voids/porosity have not been considered in the numerical simulation. Micro-CT scans or homogenization techniques to account for porosity in simulations can be incorporated in the future.

Neri et al. [33] used discarded household materials (such as cardboard, fabric, and egg cartons) to fabricate various sound-absorbing panels. The red curve corresponds to the sound absorption curve of sample 22 in their paper. This sample has a thickness of 50 mm and is made of two non-perforated spaced egg cartons whose cavities face the sound source. As shown in Figure 11b, it is found that opening holes can achieve better sound absorption at low frequencies. Inspired by owls, Wang et al. [34] combined micro-gap plates, porous fiber materials, and micro-perforated membranes to propose a multi-layer sound-absorbing structure with a total thickness of 50 mm. From the current preliminary experimental results, the structure in this paper can achieve good sound absorption effects near 1000 Hz. Moreover, the thickness of the sample in this paper is only 30 mm, which has obvious advantages in terms of size, and the structure is more compact and more flexible in design. In the future, the sound absorption effect can be further adjusted and optimized by methods such as perforating the surface and optimizing the configuration.

For uncertainties analysis, the impedance tube system errors include inherent microphone phase and amplitude calibration errors, installation positioning errors, and background noise. The impedance tube system (Model: BSWA SW422) we used has a specified amplitude measurement uncertainty of ±0.5 dB and a phase uncertainty of ±2° within its standard frequency range. The root mean square error (RMSE) between the TMM and FEM values is found to be 0.05, demonstrating good predictive accuracy of the theoretical model.

Generally, it is believed that the Archimedes spiral channel broadband absorber is effective and the agreement with theory and simulation proves the reliability of the design. Three-dimensional printing technology has significantly reduced the difficulty of preparing complex structures. The sample preparation and testing processes in this study are repeatable and scalable. Although the absorption frequency of the structure is concentrated on specific frequencies at present, this design can expand the effective frequency band by adjusting the spiral parameters (such as channel width and curvature). In specific scenarios (such as specific noise control and acoustic device integration), the efficient absorption characteristics of this structure have clear value, especially suitable for fields with limited space or requiring customized acoustic materials.

## 6. Conclusions

This study investigates acoustic metamaterials incorporating Archimedean spiral structures. Diverging from conventional resonant absorption designs reliant on cavity resonances, the proposed architecture utilizes multi-channel waveguides curved with precisely controlled geometric parameters. Its absorption characteristics arise from Fabry–Pérot (FP) resonances and viscous boundary losses within the waveguide channels.

An acoustic absorption model is developed based on the transfer matrix method and validated through numerical simulations, demonstrating strong consistency between theoretical predictions and computational results. Theoretical analysis and parametric studies revealed that the number and magnitude of absorption peaks are predominantly governed by the spiral waveguide length, where increased length induces higher-order resonant modes and broader peak distribution. Through optimization of geometric parameters, efficient broadband low-frequency absorption has been achieved.

Experimental validation is conducted using 3D-printed prototypes, confirming the accuracy of both theoretical and numerical models. Future work will focus on parallel configurations of heterogeneous unit cells to further broaden the effective absorption bandwidth and investigate coupling effects between adjacent units on collective acoustic performance.

## Data Availability

The original contributions presented in this study are included in the article. Further inquiries can be directed to the corresponding authors.
